# Hyperthermia by near infrared radiation induced immune cells activation and infiltration in breast tumor

**DOI:** 10.1038/s41598-021-89740-0

**Published:** 2021-05-13

**Authors:** Wan Fatin Amira Wan Mohd Zawawi, M. H. Hibma, M. I. Salim, K. Jemon

**Affiliations:** 1 Department of Biosciences, Faculty of Science, Universiti Teknologi Malaysia, 81310 Johor Bahru, Johor Malaysia; 2 Department of Pathology, School of Medicine, University of Otago, Dunedin, New Zealand; 3 School of Biomedical and Health Sciences, Faculty of Engineering, Universiti Teknologi Malaysia, 81310 Johor Bahru, Johor Malaysia; 4 Cancer and Infectious Diseases Group, Health and Wellness Research Alliance, Universiti Teknologi Malaysia, 81310 Johor Bahru, Johor Malaysia

**Keywords:** Cancer microenvironment, Tumour immunology

## Abstract

Breast cancer is the most common cancer that causes death in women. Conventional therapies, including surgery and chemotherapy, have different therapeutic effects and are commonly associated with risks and side effects. Near infrared radiation is a technique with few side effects that is used for local hyperthermia, typically as an adjuvant to other cancer therapies. The understanding of the use of near NIR as a monotherapy, and its effects on the immune cells activation and infiltration, are limited. In this study, we investigate the effects of HT treatment using NIR on tumor regression and on the immune cells and molecules in breast tumors. Results from this study demonstrated that local HT by NIR at 43 °C reduced tumor progression and significantly increased the median survival of tumor-bearing mice. Immunohistochemical analysis revealed a significant reduction in cells proliferation in treated tumor, which was accompanied by an abundance of heat shock protein 70 (Hsp70). Increased numbers of activated dendritic cells were observed in the draining lymph nodes of the mice, along with infiltration of T cells, NK cells and B cells into the tumor. In contrast, tumor-infiltrated regulatory T cells were largely diminished from the tumor. In addition, higher IFN-γ and IL-2 secretion was observed in tumor of treated mice. Overall, results from this present study extends the understanding of using local HT by NIR to stimulate a favourable immune response against breast cancer.

## Introduction

Breast cancer is the most common cancer among women, regardless race or ethnicity. The number of new cases increases each year with the growing of population. A variety of treatments that are available, however almost all conventional treatments, including chemotherapy and surgery, come with side effects. Hyperthermia (HT) which one of many cancer treatments available, is a therapy that uses heat to increase body temperature above the normal temperature. This could be achieved either systemically, or locally at a particular area of the body. As a non-invasive cancer treatment strategy, local HT is ideally suitable to treat superficial tumors such as neck or breast tumors^1,2^, where the temperature given is readily attainable and tolerable to patients^3,4^. Local HT can be either within the range of 39–48 °C or exceed temperature around 70–90 °C, at which different temperature could give different effects to the tumor and the adjacent normal tissues^5,6^. The main objective of local moderate HT is to kill, weaken, or sensitise the cancer cells^7^ as well as to improve the anti-tumor immunity^8^, without affecting the surrounding normal tissues^9^.

Besides inhibiting tumor growth by necrosis and apoptosis^10,11^, multiple cancer-related studies have reported the application of fever-range HT as an immunotherapeutic agent^12^. HT was reported to stimulate the activation of antigen presenting cells (APCs) by releasing the heat shock protein (Hsp) from the dying heat-stressed cancer cells^13^. In addition, these dying cells also produce exosomes that assist the maturation of APCs and increasing tumour-specific cytotoxic T lymphocyte (CTL) response^14,15^. High level of lymphocytes was also observed in lymph node of mice treated with HT, implying the augmentation of immune response by HT treatment^12^. Local HT applied at 40–43 °C also facilitates immune cells trafficking towards tumor by increasing the permeability of tumor vasculature and perfusion^6^. Furthermore, electro-HT provides favourable tumor microenvironment (TME) for immune cells to function optimally, hence would be beneficial for combination with immunotherapy^16,17^.

Despite showing some clinical benefits, HT still can be regarded as an uncommon therapy for cancer in clinical practice. This could be partly due to the inadequate monitoring of the thermal dose or tumor temperature during the treatment^18^. Furthermore, a safe and effective treatment protocol is very crucial for HT to be a candidate in clinical application. The use of near infrared radiation (NIR), one of the many approaches for local HT treatment, has been attractive to researchers and physicians. Unlike conventional radiation therapy, this technique omits the use of high energy radiation that could severely damage the normal cells^19^, thus resulting in a better outcome in cancer treatment. In fact, the irradiation is selective to cancerous tissue while avoiding the suppression of host immune response, thus enabling the host to achieve a maximum immune response^20^. However, NIR is primarily used in photothermal and photodynamic therapy which require the assistance from nanoparticles and photosensitizer^21^. Additionally, NIR has always been used as an adjuvant to other therapy including immunotherapy^22,23^. To the best of our knowledge, there are limited numbers of studies to date reporting the effect on immune cells activation and infiltration following monotherapy of NIR as a heat source, thus our study aimed to fill this gap.

This study was undertaken to confirm and extend the understanding of using local HT by NIR as a heat source in triggering immunological response. In the present study, using a subcutaneous breast tumor model, we report immune cells activation and infiltration following HT treatment that might lead to the enhanced anti-tumor response.

## Results

### Local HT by NIR reduces tumor growth in tumor-bearing mice

**Figure 1:**
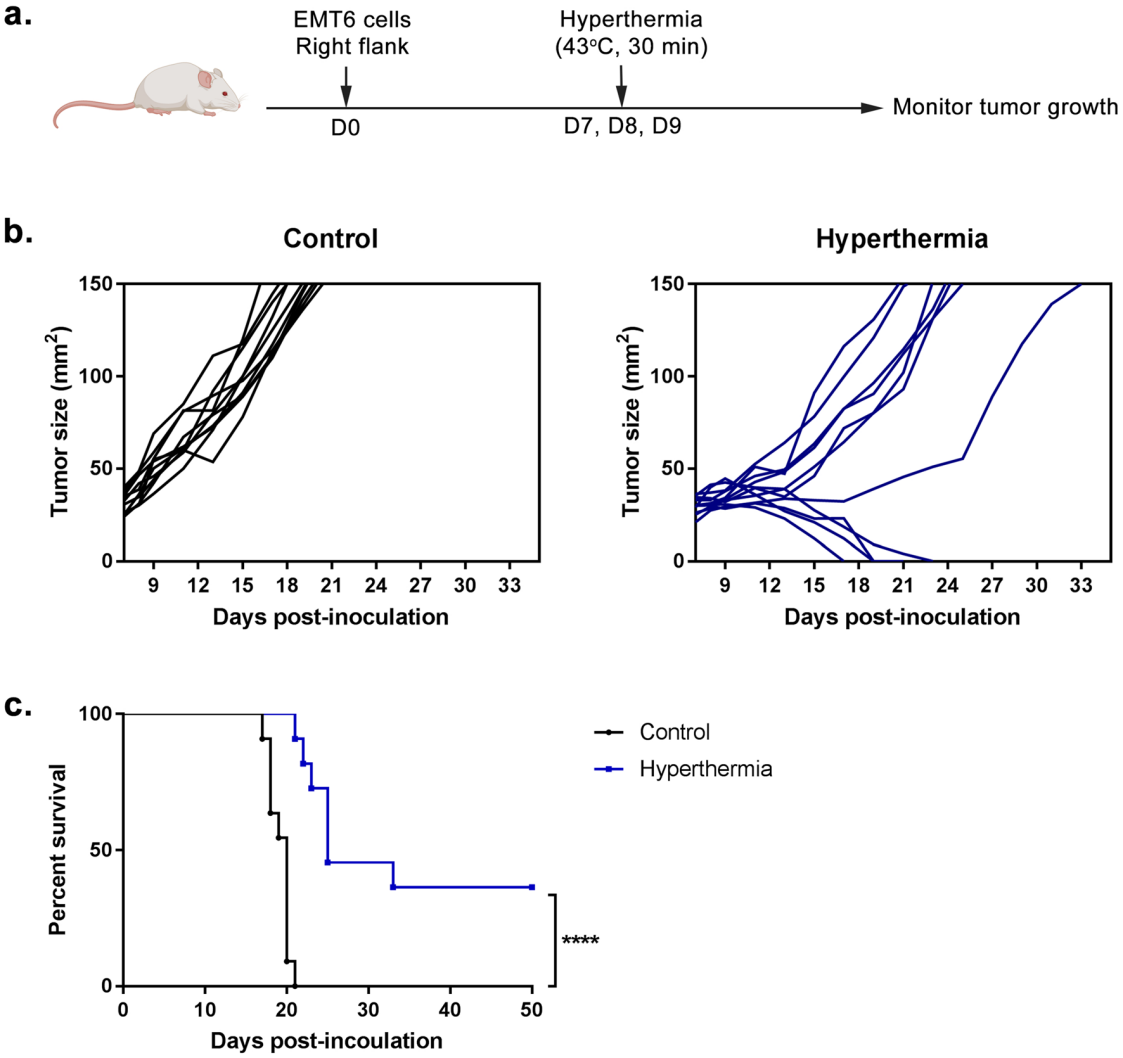
Local hyperthermia by NIR reduces tumor growth in tumor-bearing mice. (**a**) Schematic time course to study EMT6 tumor progression following treatment. (**b**) Line graph depicting the individual tumor growth in control and treated group (**c**) Survival curves of mice in 50 days after treatment. *n* = 11 per group. ****P < 0.0001 (Mantel-Cox analysis).

Mice that were injected with EMT6 cells (Fig. 1a) all had palpable sub-cutaneous tumors by day 7 post-injection, ranging from 25 to 40 mm^2^ in size. During tumor irradiation at day 7, the tumor reached 43 °C in less than 10 min and the temperature was maintained for 30 min thereafter. Figure 1b shows the tumor growth profile in the HT treatment group in comparison with untreated control group. Tumor growth in HT treated mice was delayed by 58% as compared to the tumor growth in control group. However, this data is not significant which may be due to the variation in tumor sizes between individual mice in treated group. As shown in the graph, there were two distinct tumor profile, with one showing a slow increment and the other one showing a reduction in tumor size. Four out of 11 (36%) mice in the HT treated group were tumor-free within two weeks following the treatment and remained tumor-free for 50 days thereafter before euthanized. This result was accompanied by a significant increased (*P* < 0.0001; Mantel-Cox analysis) in the median survival of tumor-bearing mice (19.5 and 26.5 for control and HT treated group, respectively) (Fig. 1c).

### Local HT by NIR reduces tumor cells proliferation

**Figure 2:**
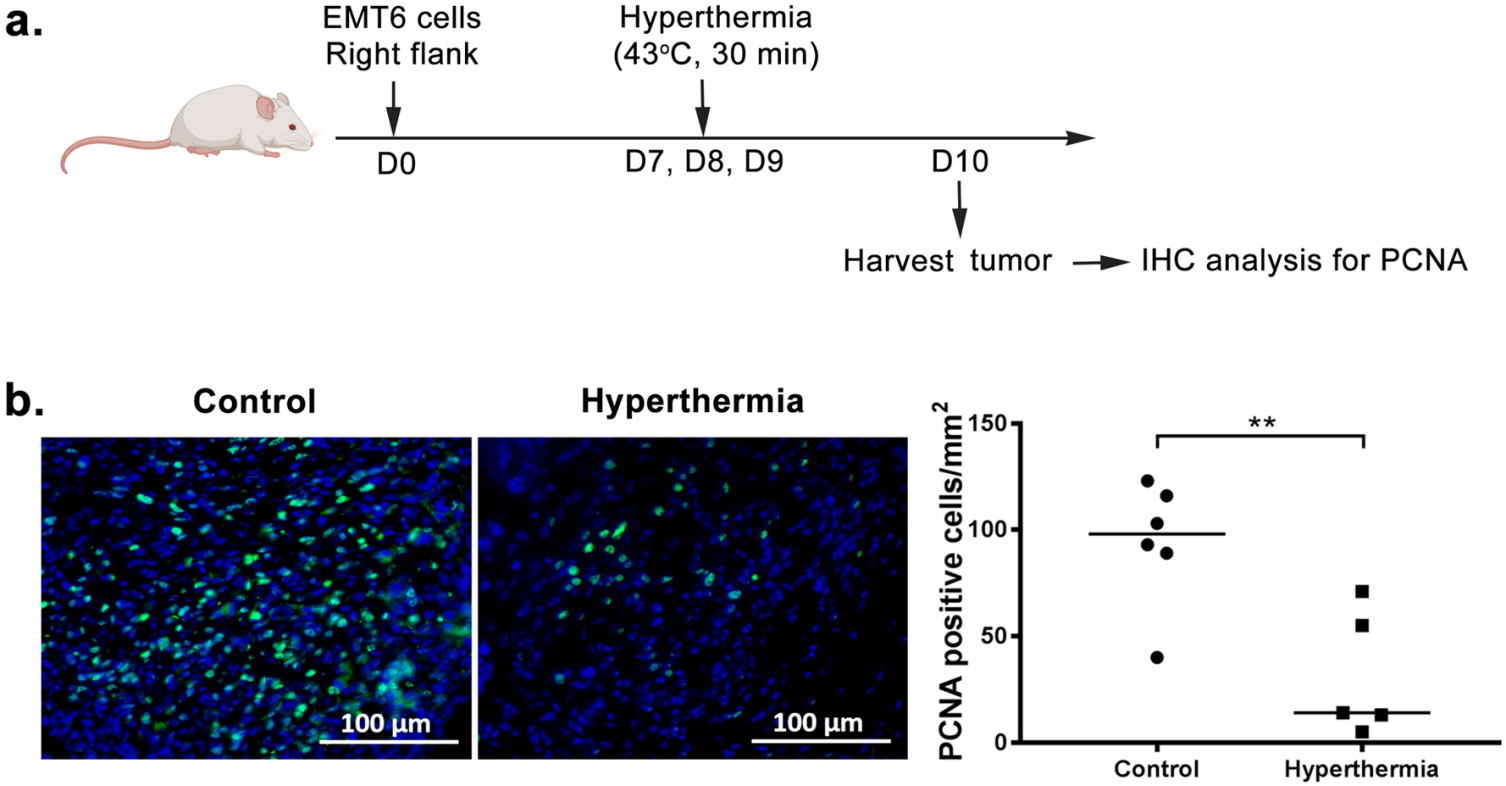
Local hyperthermia by NIR reduces tumor cells proliferation**.** Mice were sacrificed 3 days after the first treatment. (**a**) Schematic time course to examine the proliferating cells. (**b**) Fluorescence macrograph showing the expression of PCNA indicating the proliferating tumor cells and graph showing PCNA positive-stained cells per mm^2^. *n* = 5/6 per group. **P < 0.005 (Student’s *t*-test).

To investigate the effect of HT on tumor tissue, tissue was harvested and processed for immunohistochemical analysis 24 h after the last treatment (Fig. 2a). Proliferating cell nuclear antigen (PCNA) is essential for cellular processes such as DNA replication^24^ and commonly used as a marker of cell proliferation. Immunostaining of tissue section with PCNA demonstrated large numbers of actively proliferating tumor cells in the untreated group at 24 h post-treatment (Fig. 2b). Meanwhile, a reduced proportion of areas with PCNA-positive cells was observed in treated tumor. Although there were some residual cells with proliferating activity at the tumor edge, overall data confirmed the inhibition of proliferation in HT group. In addition, it was observed that the nuclei in treated tumor were larger when compared to untreated tumor, suggesting the heterogeneity across the treated tumor which may consists of other cell types including immune cells. The significant reduction (*P* < 0.005; Student’s *t*-test) of cell proliferation in the tumor tissue indicates the retardation of tumor growth. This finding was also in line with our pathological examination of the HT-treated tumor tissue, which showed prominently dead tumor cells, implying necrosis^25^ and apoptosis^26^ following HT (results not shown).

### Local HT by NIR increases tumor Hsp70 expression

**Figure 3:**
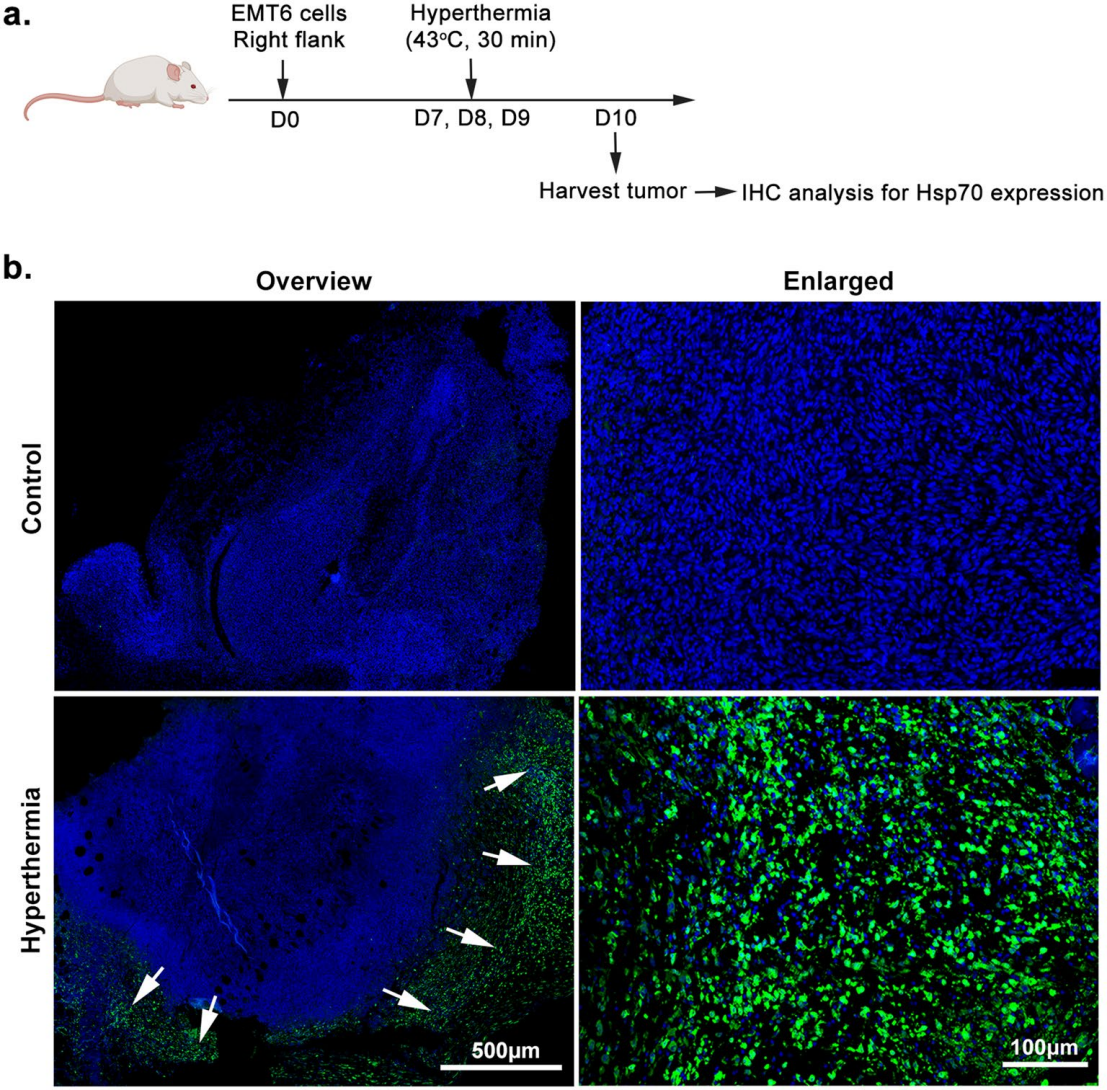
Local hyperthermia by NIR increases tumor Hsp70 expression. Mice were sacrificed 3 days after the first treatment. (**a**) Schematic time course to measure the Hsp70 expression. (**b**) Fluorescence micrograph showing the expression of Hsp70 (shown in green) in tumor. Arrow indicates the area of Hsp70 abundancy. *n* = 5/6 per group.

Within the tumor microenvironment, HT has been reported to induce heat stress to the cells. In response to this adverse stress, the expression of heat shock proteins would normally be increased^27^. We therefore wanted to examine Hsp expression in response to HT treatment. To test this, mice were injected subcutaneously with EMT6 cells, the tumor was irradiated three times at days 7, 8 and 9 post injection, and the tumor tissue was harvested one day later for immunohistochemical staining for Hsp70 (Fig. 3a). In HT-treated group, Hsp70 was strongly expressed and abundantly distributed around the necrotic area that spread along the peripheral region of the tumor (Fig. 3b). In contrast, there was no Hsp70 detected in the untreated tumor.

### Local HT by NIR increases DC activation in lymph node and tumor-infiltrated T cells

**Figure 4:**
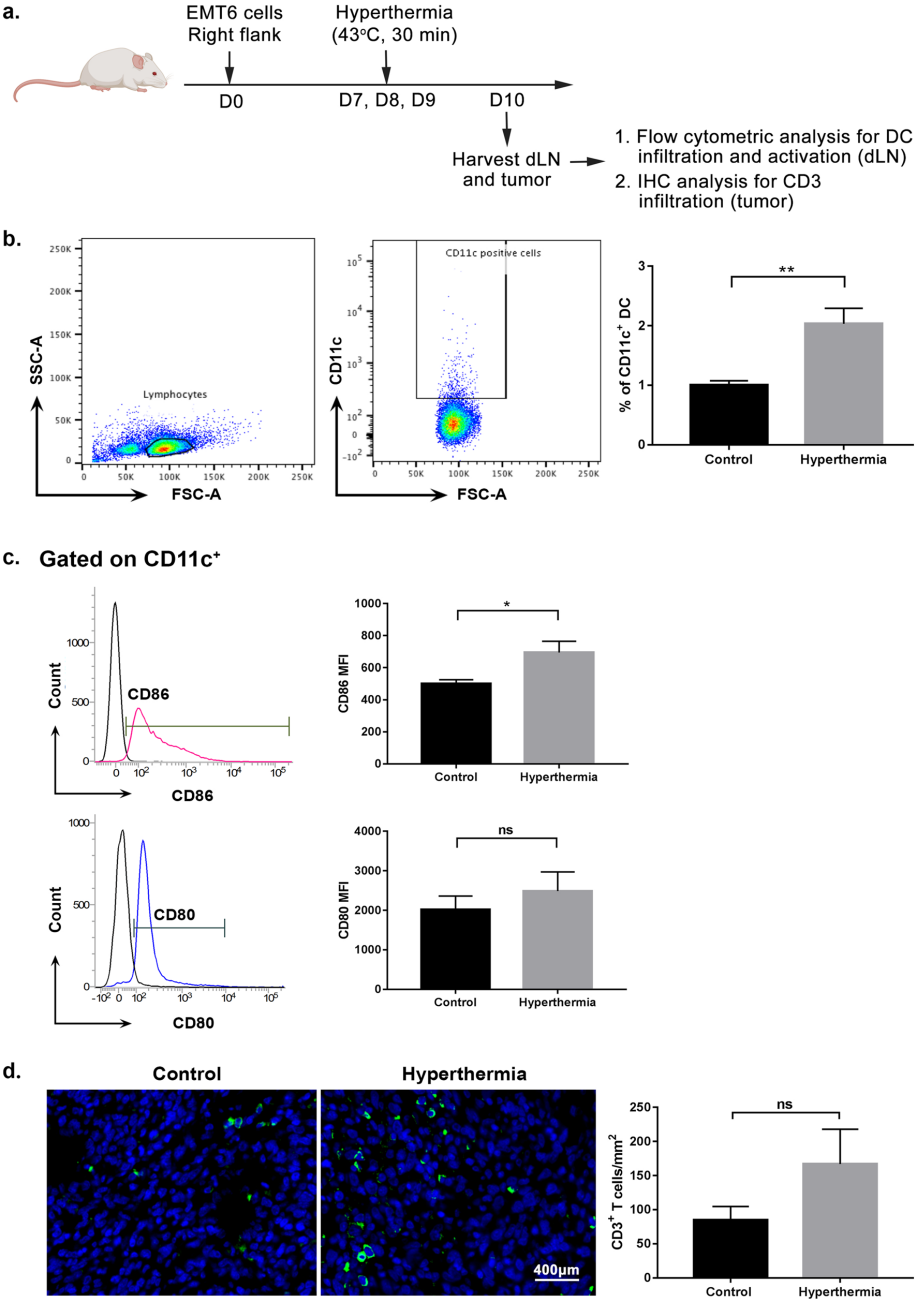
Local hyperthermia by NIR increases DC activation in lymph node and tumor-infiltrated T cells. Mice were sacrificed 3 days after the first treatment. Lymph nodes were analyzed by flow cytometric analysis while tumor was stained for immunohistochemical analysis**.** (**a**) Schematic timecourse to measure the activation of DC in dLN and the infiltration of CD3 T cells in tumor. (**b**) Dot plot showing the gating strategy of CD11c. Cells were gated based on forward and side scatter profile to exclude the doublet cells. Lymphocytes were then gated and further analyzed for the percentage of CD11c as shown in graph. DCs activation increases following hyperthermia treatment, compared to untreated group. (**c**). Graph showing Mean Fluorescence Intensity (MFI) of CD80 and CD86 expression (± mean) on CD11c^+^ cells. (**d**) CD3 T cell numbers are shown as the median of CD3 cells per mm^2^. Each point represents an individual mouse. *n* = 5/6 per group. ns-*P* > 0.05, *P < 0.05, **P < 0.005 (Student’s *t*-test).

An early step in initiation of the adaptive anti-tumor immune response is the uptake of tumor antigen by APCs, followed by the presentation to T cells of tumor associated peptides in the major histocompatibility (MHC) I or MHC II complex. As a key player in the adaptive immune response, we evaluated the number of activated CD11c^+^ dendritic cells in dLN at 24 h after the last HT treatment (Fig. 4a). Cells were gated based on forward and side scatter profile to exclude the doublet cells. Lymphocytes were then gated and further analyzed for the presence of CD11c. The activation and maturation of DCs was also determined by measuring the expression of co-stimulatory molecules CD80 and CD86 on the positive CD11c cells. There was a significant increased (*P* < 0.005; Student’s *t*-test) in the number of CD11c^+^ cells in the tumor dLN of the HT treated group when compared to the control group (Fig. 4b). In addition, expression of CD86 and CD80 was increased in the HT treated group, as compared with the control group, implying higher DCs activation in lymph node following HT by NIR (Fig. 4c).

The infiltration of CD3^+^ T cells in tumor has been used to predict prognosis in cancer and is associated with active recruitment of T cells responsible for cell death^28,29^. We enumerated the number of CD3^+^ T cells that infiltrated the tumor by staining with an anti-CD3 antibody. Our result from immunohistochemical staining showed a non-significant increase of CD3^+^ T cells in heated tumor, which suggests that T cells have been activated by mature DCs and trafficked into the tumor (Fig. 4d).

### Local HT by NIR increases CD8 but not CD4 infiltration into EMT6 breast tumor

**Figure 5:**
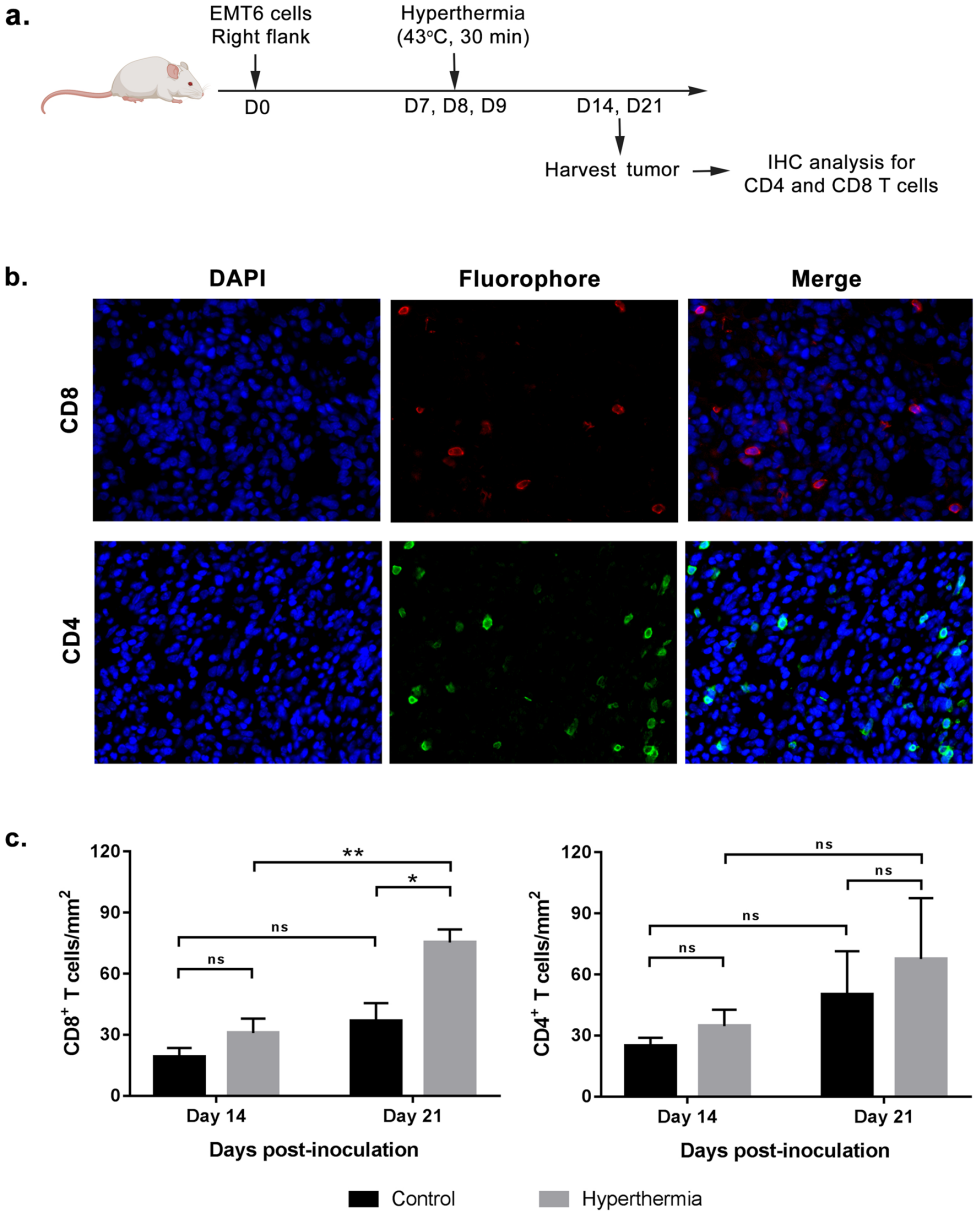
CD8 and CD4 T cells infiltrated in tumor increases over time. Mice were sacrificed at two time points after the treatment; day 14 and day 21 post-inoculation. Tumor were harvested and paraffin-fixed for staining with anti-CD4 and ant-CD8 antibody. (**a**) Schematic time course to measure the number of tumor–infiltrated CD4^+^ and CD8^+^ cells by immunohistochemical analysis. (**b**) Representative fluorescence images of stained tumor. (**c**) CD4^+^ T and CD8^+^ cell numbers are shown as the median of cells per mm^2^. Each point represents an individual mouse. *n* = 3–6 per group. ns-*P* > 0.05, *P < 0.05, **P < 0.005 (ANOVA).

**Figure 6:**
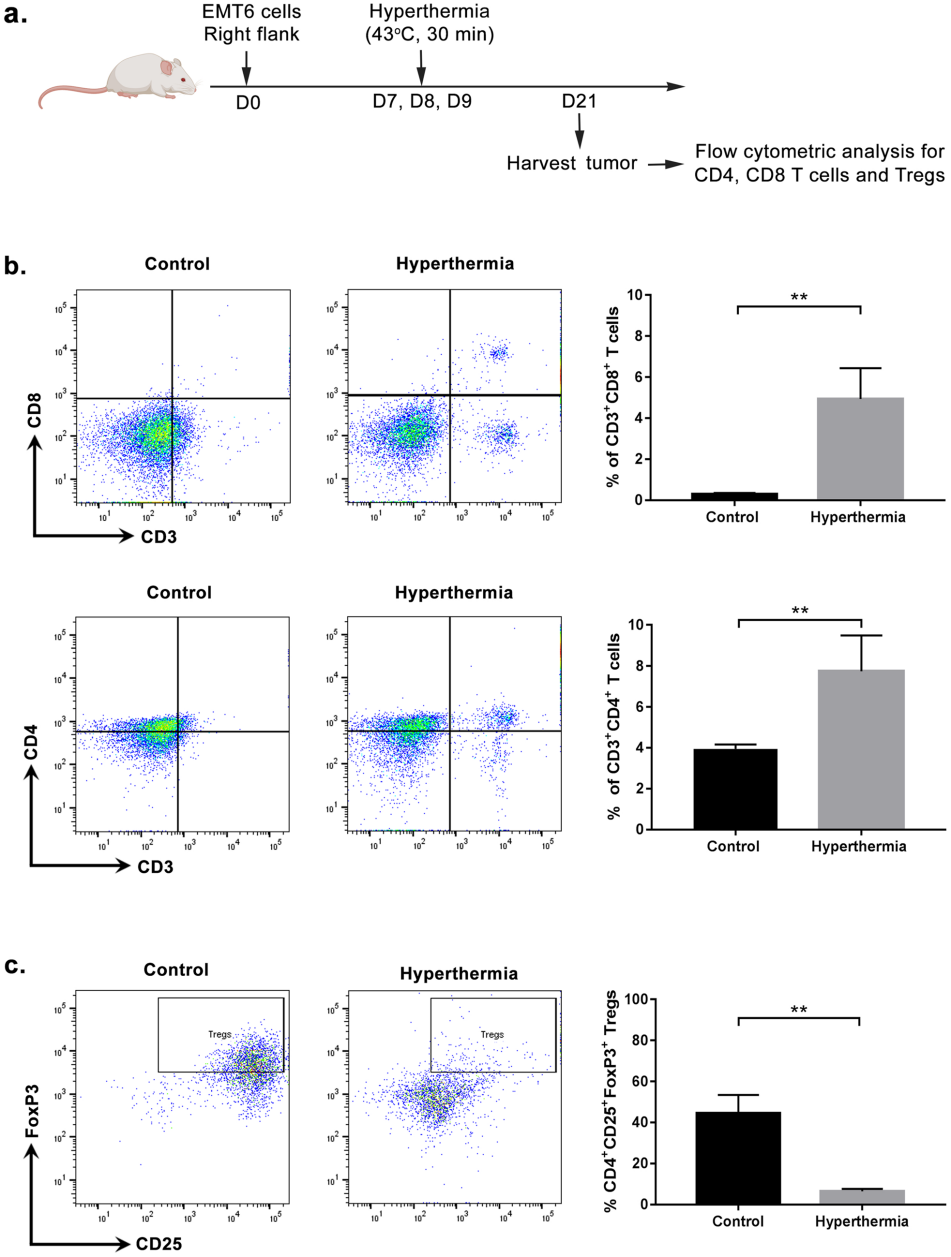
Influence of local hyperthermia by NIR on T cells. Mice were sacrificed 14 days after treatment to evaluate T cells infiltrated at tumor site. Single cells suspension was analyzed by flow cytometric analysis. (**a**) Schematic timecourse to study the tumor-infiltrated T cells. (**b**) Representative dot plot showing population of CD4 and CD8 T cells infiltrated in tumor. Graph showing percentage of CD3^+^CD8^+^ and CD3^+^CD4^+^ T cells (± mean). (**c**) Representative dot plot showing population of Tregs infiltrated in tumor. Gated CD4^+^ cells were measured for co-expression of CD25 and FoxP3. Graph showing the percentage of CD4^+^CD25^+^FoxP3^+^ cells (± mean). *n* = 5 per group. ns-*P* > 0.05, **P < 0.005 (Mann–Whitney test).

To elucidate CD4 helper T cell and CD8 cytotoxic T cell infiltration into the tumor following HT treatment, tumor tissue was harvested at day 14 and 21 post-inoculation and immunohistochemical analysis was carried out (Fig. 5a). Figure 5b shows that HT had little effect on the number of tumor infiltrated CD4^+^ T cells, with only a slight difference observed between both groups (Fig. 5c). On the other hand, the number of CD8^+^ T cells showed a significant increased on treated tumor at both timepoints, with higher CD8^+^ T cells number was observed at day 21. This result was further validated using flow cytometric analysis (Fig. 6a and Supplementary Fig. S1). As expected, there was significant difference (*P* < 0.005; Student’s *t*-test) between both groups, with treated group shows higher percentage of CD3^+^CD8^+^ T cells as compared to control (Fig. 6b).

### Local HT by NIR inhibits regulatory T cells infiltration in tumor

We investigated the effect of NIR on the immunosuppressive cells, particularly regulatory T cells (Tregs). Tregs is a sub-population of T cells, described as expressing CD4, CD25, and FoxP3. Tregs infiltrating the tumor was examined at day 21 post-inoculation. Flow cytometric analysis in Fig. 6c shows a low number of CD4 T cells co expressing CD25 and FoxP3 in NIR-treated tumor, at around 80% reduction as compared to control. The percentage of CD4^+^CD25^+^FoxP3^+^ Tregs among the total tumor-infiltrated CD4^+^ cells is 44.45 ± 9.03 in control group as compared to 6.47 ± 1.18 in NIR treated group (*P* < 0.005; Student’s *t*-test). In light of the notable CD8 T cells infiltrate in the NIR samples, we also calculated the ratio of CD8 to Tregs that had infiltrated the tumor. The median of CD8:CD4^+^CD25^+^FoxP3^+^ Tregs was significantly higher in NIR treated group as compared to control (0.0076 and 0.91 at *P* = 0.0286, respectively). This result is consistent with a good prognosis, as shown by Preston et al*.* (2013), who reported a higher CD8:Treg ratio in ovarian cancers that had a good prognosis. The suppression of immune response by Tregs is often linked to poor naïve T cell proliferation^30,31^. Therefore, depletion of Tregs shown in this experiment supports the results described in Fig. 6b that show a high number tumor-infiltrated CD8 T cells, suggesting the enhancement of CD8 T-cell effector function in eradicating tumor.

### Effect of local HT by NIR to intratumoral cytokine

**Figure 7:**
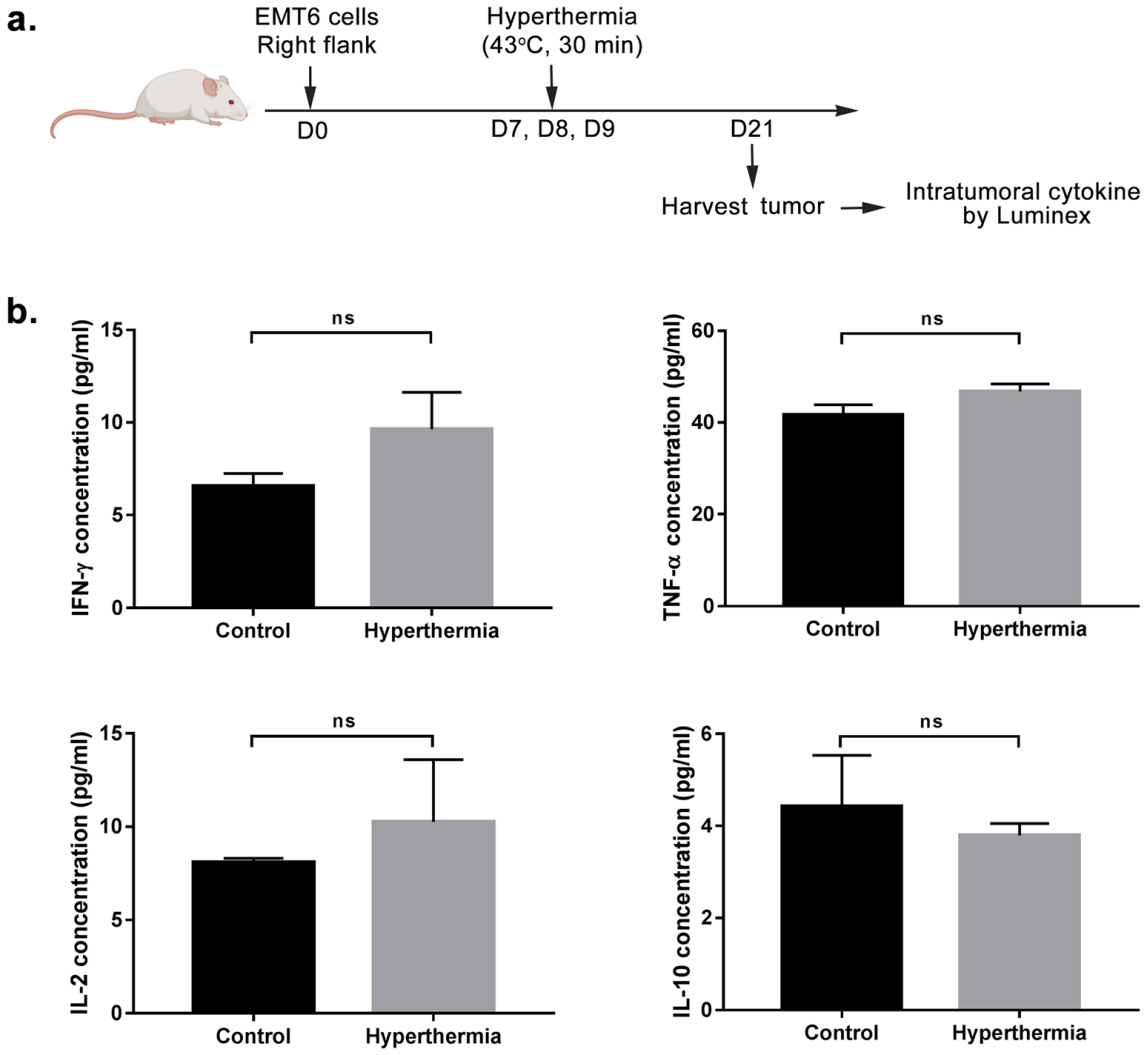
Effect of local HT by NIR on intratumoral cytokine. Mice were sacrificed 2 weeks after the first treatment. Tumor were harvested and total protein concentration were measured. The level of intratumoral cytokine IFN-γ, TNF-α, IL-2, and IL-10 were then analyzed by Luminex. Bar graph showing **(a)** IFN-**γ, (b)** TNF-α, **(c)** IL-2 and **(d)** IL-10 concentration (± mean). *n* = 5/6 per group. ns-*P* > 0.05 (Student’s *t*-test).

To further understand the effect of NIR on the immune related molecules, we next measured intratumoral cytokines including IFN-γ, TNF-α, IL-2 and IL-10 at 21 days post-inoculation (Fig. 7a). These cytokines were selected as they play a diverse role in breast cancer progression and adaptive immunity. Tumor was measured for total protein concentration and analyzed by Luminex technology for cytokine concentration. Our findings demonstrated a non-significant increase of IFN-γ in tumor of treated mice compared to untreated mice (Fig. 7b). Besides, IL-2 level in HT treated tumor displayed a slight increase, while the concentrations of TNF-α and IL-10 showed no obvious changes as compared to control group.

## Discussion

Local HT treatment has been widely used to treat solid tumors in experimental models^32–34^ and in cancer patients^35–38^. Heat may come from various external sources including infrared radiation, radiofrequency, microwave and ultrasound, where these approaches could give different effects in eradicating the tumor. Potentially, NIR was reported to be efficient in killing the tumor cells selectively without causing disruption on surrounding normal tissues. In this study, heat was applied using wIRA with a 750-W halogen lamp and a 780 nm high pass filter, resulting a peak output at 820 nm. This device is commonly used to treat skin diseases, wound and many other areas of medicines. WIRA is easy to operate, gentle to skin and allows deeper penetration compared to conventional infrared therapy^39^. As breast cancer is a superficial tumor, the use of local HT is clinically appropriate and has potential to treat the tumor effectively. While conducting the local HT procedure, it is very important to monitor the tumor temperature precisely because even the slightest temperature change could give a different level of heat-stress to the tumor and stromal cells. The treatment efficacy could also be temperature dependent. For instance, heating tumor at 42 °C may improve vasculature permeability thus better immune cells trafficking into the tumor^6^. This scenario however, was not observed at temperature more than 43 °C due to hemorrhage^6^. Meanwhile, heating the tumor at 45 °C or within the range of 70–90 °C, at which the goal is to ablate the tumor cells, may have risk of insufficient heating which could cause epithelial-mesenchymal transition (EMT)^40^. Recent studies have also reported the promotion of EMT following incomplete ablation using RFA or ionizing radiation^41,42^. This adverse effect is thought to be due to the upregulation of flotilin protein in residual cells that promotes tumor invasion which led to tumor progression and metastasis^41^. NIR technique used in this study however, may reduce an EMT possibility since the temperature used is within the fever-range temperature that is believed to be immunotherapeutic^6^. We selected 43 °C (mild hyperthermia) for our HT approach and the treatment was repeated three times for 30 min each to ensure the optimum effect was achieved.

Using this approach, HT was able to inhibit the growth of subcutaneous EMT6 tumor, with 36% of treated mice showing complete tumor regression. These mice also survived for 50 days after the treatment, demonstrating that HT could provide a therapeutic benefit that prolongs mice survival. HT caused tumor cells to undergo necrosis and apoptosis which affects the cell number and tumor size and subsequently led to inhibition of tumor growth. Consistent with tumor inhibition, HT also decreased cell proliferation, which was observed through the reduction of PCNA expression. Therefore, we reasoned that NIR not only induced cell death but also retarded the division of new cells by arresting the cell cycle. In particular, NIR induced heat stress to the cells. It was reported that the stress response pathway always elicits cell cycle arrest to circumvent the transmission of damaged macromolecules and provides the time for cells to repair themselves, resulting in suppression of cell proliferation^43^. Accordingly, a more pronounced proliferating activity was found in our untreated breast tumor, which correlates with the findings of other clinical studies that demonstrated PCNA is greatly involved in breast cancer initiation and development^44,45^. However, it is interesting to investigate whether NIR could lead to the tumor inactivation. This could be done either by tumor rechallenged or inoculating tumor at a distal site, as exemplified in melanoma and pancreatic tumor model^12,46,47^, thus future study could focus on these two approaches.

Heat shock proteins have been recognized as an important participant in immune system^27,48^. Hsps are present in normal cells conditions but will be highly expressed under stress condition including heat-stress. Necrotic cancer cells destroyed by HT will release antigenic tumor peptides that includes Hsp into extracellular matrix. These extracellular Hsps then will act as danger signal to activate dendritic cells and subsequently T cells to generate adaptive immune response. The antitumor effect is dominated by Th1 CD4^+^ T lymphocytes. Generally, CD4^+^ T cells perform immunosurveillance and regulate immune system, alongside with Th1 and Th2 cytokines to balance the cell-mediated immunity^49^. Th1 CD4^+^ T lymphocytes secretes Th1 cytokines including IFN-γ, TNF-α, IL-2 which in turn act as marker for Th1 immune response. In response to cancer, CD4^+^ T cells provide help in the priming of tumor-specific CD8^+^ T cells, thus permitting the differentiation and expansion of tumor antigen-specific CTLs^50^. Subsequently, effector CTLs migrate into the tumor and perform their killing activity accordingly^51^. The major role of CD8 T cells in tumor inhibition has been established in tumor treated with CD8-depleting antibodies where the tumor was unable to induce resistance to tumor rechallenged, thus indicating that CD8^+^ T cells are required for treatment efficacy^12^. Findings from this study also demonstrated a significant increased of tumor infiltrated-CD8^+^ T cells with greater CD8^+^ T cells infiltrates tumor over time. This scenario suggests HT treatment likely stays effective for a longer period, which may be related to higher antigen-specific CTLs produced as a result from increased Hsp-peptide complex cross presentation by DCs ^52^. The cross priming of antigen-specific CTL is however not directly shown in this study, but the overall result of tumor inhibition could speculate the antigen-specific CTLs. Therefore, future study is needed to confirm the tumor-specific immunoreaction for example by using adoptive transfer of NIR-activated CTLs or by co-culturing splenocytes from NIR-treated mice with different types of tumor cells to determine CTLs cytolytic activity^53^.

The upregulation of IFN-γ in tumor also supports these findings, where IFN-γ is able to inhibit tumor cell proliferation and differentiation^50^. Besides orchestrating the innate immune response, IFN-γ, which is mainly produced by activated T cells and NK cells, also mediates the adaptive anti-tumor response. For instance, IFN-γ increases the expression of MHC, making them more susceptible to immune recognition and destruction by CD8^+^ T cells^54^. Other than IFN-γ, IL-2 also plays a critical role in the induction of the immune response. However, the non-significant increase in IL-2 reported here maybe due to the dual role played by this cytokine. IL-2 is responsible for regulating CD8^+^ T cells effector function as well as promoting the differentiation of CD4^+^ T lymphocytes into Tregs. On another note, these intratumoral cytokines were analyzed 2 weeks post-treatment, which is considerably late as they usually secreted at the early stage to help initiate the immune response. This may also explain the non-significant different in expression between both groups. Hence, future study is suggested to be conducted at day 4–7 post-treatment.

Tregs have long been known to suppress the immune response and favor the growth of tumor cells, causing the cancer to progress and to become metastatic. The recruitment of CD4^+^CD25^+^FoxP3^+^ T cells to the tumor distorts T cells responses from an effector to a regulatory subtype, causing protective antitumor immunity to diminish^55^. As been reviewed by Plitas and Rudensky (2020), the presence of this population in tumor microenvironment is linked with the suppression of T cells, NK cells and DC in animal models and in cancer patients^56^. Similar situations observed in our data correlate with those findings, suggesting the ability of NIR in limiting the immunosuppressive activity. The CD8:Treg ratio is commonly used to predict prognosis in breast cancer ^57,58^. NIR also causes higher CD8:Treg ratios at D21 post-inoculation, which implies a favorable prognosis. In conjunction with these results, HT is believed to disrupt the function of Tregs and interfere with their migratory properties^59^. Taken together, these findings also add to the speculation that NIR is able to dampen the immunosuppressive activity of Tregs.

Collectively, our data suggest that a monotherapy of NIR is able to delay tumor progression and promote the activation and infiltration of immune cells in breast tumor. With easy administration in a controlled manner, the application of local hyperthermia could potentially offer clinical benefit for the treatment of breast cancer.

## Methods

### Cell line

The mammary carcinoma cell line EMT6 (ATCC CRL-2755) was maintained in Waymouth’s MB medium (Life Technologies), supplemented with 10% heat inactivated fetal bovine serum (FBS; Life Technologies) and 1% penicillin/streptomycin (100 U/ml penicillin and 100 μg/ml streptomycin, Sigma).

#### In vivo tumor model

All animal experiments were performed under the approved protocol by the Universiti Kebangsaan Malaysia Animal Ethics Committee (Approval code: UTM/2016/KHAIRUNADWA/28-JAN./729-FEB.-2016-JAN.2019). This study was conducted in accordance with the ARRIVE and relevant guidelines. Female Balb/c mice (8—10 weeks of age) weighing between 18 and 25 g were purchased from Universiti Kebangsaan Malaysia, Bangi, Malaysia. Mice were anaesthetized by subcutaneous injection of a cocktail of Ketamine/Xylazine/Zoletil (KTX) at dosage of 0.1 ml/25 g body weight. Mice were inoculated with 5 × 10^5^ EMT6 cells in 100 μl phosphate-buffered saline (PBS, pH 7.4) into the right flank subcutaneously.

### Local HT treatment

At day 7 post-EMT6 inoculation, tumors of about 30 mm^2^ in size were subjected to local HT treatment by means of infrared radiation using a water-filtered infrared irradiator (wIRA, Hydrosun 750, Germany). A 30-gauge thermocouple was inserted into tumor center to precisely monitor the tumor temperature. For the irradiation, mice were covered by a circular shield, exposing only the tumor site. The tumor was heated to 43 °C, and the temperature was maintained by continuous regulation of the heat source that switched on and off intermittently for 30 min, controlled by the inserted thermocouple. This treatment was repeated for three times, every consecutive day. Tumor size was monitored by measuring the tumor using a digital caliper at every alternate day.

### Immunohistochemical analysis

Tumors were harvested at day 10, 14 and 21 post-treatment. Tumor tissues were fixed in 10% neutral-buffered formalin and embedded in paraffin for staining. Formalin-fixed paraffin-embedded (FFPE) tissue Sects. (4 μm) were deparaffinized in xylene followed by rehydration with a series of alcohol washes. For antigen retrieval, tissue sections were heated for 20 min in 10 mM sodium citrate buffer (pH 6.0) or Tris/5% Urea (pH 9.5). Sections were blocked with 5% skim milk in Tris-buffered saline (TBS) and stained with primary antibodies overnight at 4 °C. Rabbit anti-mouse PCNA (clone C19, Abcam), mouse anti-mouse Hsp70 (W27, Santa Cruz), rabbit anti-mouse CD3 (SP7, Abcam), rat anti-mouse CD8 (4SM15, eBioscience) and rabbit anti-mouse CD4 (EPR19514, Abcam) were used for primary staining. The next day, sections were incubated with secondary antibody for 30 min with the addition of 1:50 DAPI after the first half an hour. Alexa Fluor 488 goat anti-rabbit IgG (1:500, Invitrogen) and Alexa Fluor 594 goat anti-rat (1:200, abcam) and Alexa Fluor 488 anti-mouse IgG (1:200, Invitrogen) were used for secondary staining. Each step was followed by 3 times of washing step, 5 min each with TBS at RT. Coverslips were applied to the slides using SlowFade Diamond (ThermoFisher, USA).

Sections were viewed using fluorescence microscope (Olympus, France). For PCNA staining, a minimum of five fields were randomly selected throughout the tumor and images were captured to enumerate the positive-stained cells. Meanwhile for CD3, CD4 and CD8, enumeration was performed for the whole tumor tissue. Positive-stained cells were quantified using ImageJ software (https://imagej.nih.gov/ij/).

### Flow cytometric analysis

Draining lymph node (dLN) and tumor tissues were harvested at day 10 or day 21 post-inoculation, respectively. To extract cells from dLN, the dLNs were excised and immediately placed in cold RPMI media (Life Technologies). The dLN was minced, filtered through a 40 μm strainers and washed at 300 × *g* for 5 min.

To extract cells from tumor tissue, the tissue was excised and cut into small fragments (1-4 mm) then incubated for 30 min at 37 °C in RPMI media containing 1 ml of tumor digestion medium (10 U/ml of Collagenase I, 400 U/ml of Collagenase IV and 30 U/ml of DNAse) (Worthington Biochemical, USA) and 1 ml of Non-Enzymatic Cell Dissociation Buffer (Sigma). The tissue was minced, filtered through a 40 μm strainers and washed at 300 × *g* for 5 min. In order to remove red blood cells in tumor cell suspension, the pellet was resuspended in 5 ml erythrocyte lysis buffer for 10 min at RT, then the cells were washed again.

For the cell staining for flow cytometry, cell suspensions were resuspended in FACS buffer to achieve 1–2 × 10^7^ cells/ml and blocked with anti-CD16/32 (BD Bioscience). For intracellular staining, cells were permeabilized and fixed using Intracellular Fixation and Fix & Perm Cell Fixation and Permeabilization kit (Invitrogen, MD, USA) according to manufacturer’s instructions.

Staining was performed by using antibodies against CD11c (clone N418, Biolegend), CD80 (16-10A, Biolegend), CD86 (GL-1, Biolegend), CD45 (30-F11, Biolegend), CD3 (145-2C11, Biolegend), CD8 (53–6.7, BD Biosciences), CD4 (GK1.5, Biolegend), CD25 (3C7, Biolegend), CD49b (DX5, Biolegend), CD19 (6D5, Biolegend), and FoxP3 (MF-14, Biolegend). Propium iodide was added at a final concentration of 1 μg/ml for 5 min prior analysis to discriminate dead cells. The data was acquired by FACSVerse with FACSuite software (BD Biosciences) and analysed using FlowJo software package.

### Intratumoral cytokine concentration

Tumor were harvested and homogenized in RIPA buffer containing 1% of protease inhibitor cocktail (Abcam) for every 100 mg of tissue. The homogenates were collected and centrifuged at 14 000 × *g* for 10 min at 4 °C. Bradford Assay kit (Bio-Rad, US) was used to quantify the total protein concentration in tumor tissue homogenates Tissue homogenates samples were analyzed to study the concentration of cytokine and chemokine (IL-10, IL-2, TNF-α, IFN-γ and MIP-1a) by using a Milliplex Map Mouse Cytokine/Chemokine Magnetic Bead Panel (MerckMillipore, Germany) using a Luminex technology. The protocol was conducted according to manufacturer’s instructions.

### Statistical analysis

Comparisons in survival between experimental groups were analyzed using Mantel-Cox analysis. Where appropriate, Mann-Whitey U Test and *t*-test were used to compare median and mean of differences in number of positive cells. ANOVA was used to compare means between groups at different timepoints. Differences were considered significant at *P* value of less than 0.05. All statistical analyses were carried out within GraphPad Prism version 7.0 (GraphPad Software, CA, USA).

## Supplementary Information

Supplementary Information.

## References

[1] Zagar TM (2010). Hyperthermia for locally advanced breast cancer. Int. J. Hyperthermia.

[2] Gao S, Zheng M, Ren X, Tang Y, Liang X (2016). Local hyperthermia in head and neck cancer: Mechanism, application and advance. Oncotarget.

[3] Dewhirst MW, Vujaskovic Z, Jones E, Thrall D (2005). Re-setting the biologic rationale for thermal therapy. Int. J. Hyperthermia.

[4] Wu F (2007). ‘Wide local ablation’ of localized breast cancer using high intensity focused ultrasound. J. Surg. Oncol..

[5] Den Brok MHMGM (2004). In situ tumor ablation creates an antigen source for the generation of antitumor immunity. Cancer Res..

[6] Toraya-brown S, Fiering S (2014). Local tumour hyperthermia as immunotherapy for metastatic cancer. Int. J. Hyperth..

[7] Tang, F., Zhang, Y., Zhang, J., Guo, J. & Liu, R. Assessment of the efficacy of laser hyperthermia and nanoparticle-enhanced therapies by heat shock protein analysis. *AIP Adv.* **4**, (2014).

[8] Rao W, Deng Z-S, Liu J (2010). A review of hyperthermia combined with radiotherapy/chemotherapy on malignant tumors. Crit. Rev. Biomed. Eng..

[9] Hegyi, G., Szigeti, G. P. & Szász, A. Hyperthermia versus Oncothermia: Cellular Effects in Complementary Cancer Therapy. *Evidence-Based Complement. Altern. Med.* **2013**, 672873 (2013). 10.1155/2013/672873 PMC3638606 23662149

[10] Pawlikowska M, Jędrzejewski T, Piotrowski J, Kozak W (2016). Fever-range hyperthermia inhibits cells immune response to protein-bound polysaccharides derived from Coriolus versicolor extract. Mol. Immunol..

[11] Happonen E (2020). Thermal dose as a universal tool to evaluate nanoparticle-induced photothermal therapy. Int. J. Pharm..

[12] Toraya-Brown S (2014). Local hyperthermia treatment of tumors induces CD8+ T cell-mediated resistance against distal and secondary tumors. Nanomed. Nanotechnol. Biol. Med..

[13] Pockley, A. G. & Henderson, B. Extracellular cell stress (Heat shock) proteins—immune responses and disease: An overview. *Philos. Trans. R. Soc. B Biol. Sci.* **373**, (2018). 10.1098/rstb.2016.0522 PMC5717522 29203707

[14] Chen T, Guo J, Yang M, Zhu X, Cao X (2011). Chemokine-containing exosomes are released from heat-stressed tumor cells via lipid raft-dependent pathway and act as efficient tumor vaccine. J. Immunol..

[15] Zhong H (2011). Induction of a tumour-specific CTL response by exosomes isolated from heat-treated malignant ascites of gastric cancer patients. Int. J. Hyperth..

[16] Tsang YW (2015). Improving immunological tumor microenvironment using electro-hyperthermia followed by dendritic cell immunotherapy. BMC Cancer.

[17] Krenacs T (2020). Modulated electro-hyperthermia-induced tumor damage mechanisms revealed in cancer models. Int. J. Mol. Sci..

[18] I *et al.* Hyperthermia: Cancer Treatment and Beyond. *Intech***i**, 13 (2012).

[19] Obayashi T (2015). Treatment with near-infrared radiation promotes apoptosis in pancreatic cancer cells. Oncol. Lett..

[20] Dees C, Harkins J, Petersen MG, Fisher WG, Wachter EA (2002). Treatment of murine cutaneous melanoma with near infrared light. Photochem. Photobiol..

[21] Dou, Q. Q., Teng, C. P., Ye, E. & Loh, X. J. Effective near-infrared photodynamic therapy assisted by upconversion nanoparticles conjugated with photosensitizers. *Int. J. Nanomedicine* 419–432 (2015). 10.2147/IJN.S74891 PMC4294652 25609954

[22] Guo L (2014). Combinatorial photothermal and immuno cancer therapy using chitosan-coated hollow copper sulfide nanoparticles. ACS Nano.

[23] Nagaya T (2015). Near infrared photoimmunotherapy targeting EGFR positive triple negative breast cancer: Optimizing the conjugate-light regimen. PLoS ONE.

[24] Strzalka W, Ziemienowicz A (2011). Proliferating cell nuclear antigen (PCNA): A key factor in DNA replication and cell cycle regulation. Ann. Bot..

[25] Li J-Y (2012). The chemokine receptor CCR4 promotes tumor growth and lung metastasis in breast cancer. Breast Cancer Res. Treat..

[26] Mantso T (2018). Hyperthermia induces therapeutic effectiveness and potentiates adjuvant therapy with non-targeted and targeted drugs in an in vitro model of human malignant melanoma. Sci. Rep..

[27] Pockley G (2003). Heat shock proteins as regulators of the immune response. Lancet.

[28] Zhou C (2018). Density and location of CD3+ and CD8+ tumor-infiltrating lymphocytes correlate with prognosis of oral squamous cell carcinoma. J. Oral Pathol. Med..

[29] Yu A (2018). Presence of lymphocytic infiltrate cytotoxic T lymphocyte CD3+, CD8+, and immunoscore as prognostic marker in patients after radical cystectomy. PLoS ONE.

[30] Gough MJ (2008). OX40 agonist therapy enhances CD8 infiltration and decreases immune suppression in the tumor. Cancer Res..

[31] Dowling MR (2018). Regulatory T cells suppress effector T cell proliferation by limiting division destiny. Front. Immunol..

[32] Tang J-C, Shi H-S, Wan L-Q, Wang Y-S, Wei Y-Q (2013). Enhanced antitumor effect of curcumin liposomes with local hyperthermia in the LL/2 model. Asian Pacific J. Cancer Prev..

[33] Datta NR (2015). Local hyperthermia combined with radiotherapy and- / or chemotherapy : Recent advances and promises for the future. Cancer Treat. Rev..

[34] Skandalakis GP (2020). Hyperthermia treatment advances for brain tumors. Int. J. Hyperth..

[35] Vargas-roig LM, Gago FE, Tello O, Aznar JC, Ciocca DR (2015). Heat shock protein expression and drug resistance in breast cancer patients treated with induction chemotherapy. Int. J. Cancer.

[36] Linthorst M (2015). Local control rate after the combination of re-irradiation and hyperthermia for irresectable recurrent breast cancer: Results in 248 patients. Radiother. Oncol..

[37] Datta NR, Stutz E, Gomez S, Bodis S (2019). Efficacy and safety evaluation of the various therapeutic options in locally advanced cervix cancer: a systematic review and network meta-analysis of randomized clinical trials. Int. J. Radiat. Oncol. Biol. Phys..

[38] Franckena M (2008). Long-term improvement in treatment outcome after radiotherapy and hyperthermia in locoregionally advanced cervix cancer: an update of the dutch deep hyperthermia trial. Int. J. Radiat. Oncol. Biol. Phys..

[39] Notter M, Germond JF, Wolf E, Berz R, Berz JP (2011). Thermography guided irradiation using water-filtered infrared-A (wIRA) and radiotherapy on recurrent breast cancer - First experiences and temperature analysis. Thermol. Int..

[40] Zhou T (2020). Insufficient radiofrequency ablation promotes epithelial-mesenchymal transition mediated by interleukin-6/signal transducer and activator of transcription 3/Snail pathway in the H22 cells. J. Cancer Res. Ther..

[41] Zhang N (2019). Insufficient radiofrequency ablation promotes the metastasis of residual hepatocellular carcinoma cells via upregulating flotillin proteins. J. Cancer Res. Clin. Oncol..

[42] Shi L (2019). Inflammation induced by incomplete radiofrequency ablation accelerates tumor progression and hinders PD-1 immunotherapy. Nat. Commun..

[43] Chircop M, Speidel D (2014). Cellular stress responses in cancer and cancer therapy. Front. Oncol..

[44] Juríková M, Danihel Ľ, Polák Š, Varga I (2016). Ki67, PCNA, and MCM proteins: Markers of proliferation in the diagnosis of breast cancer. Acta Histochem..

[45] Qiu X (2017). Correlation analysis between expression of PCNA, Ki-67 and COX-2 and X-ray features in mammography in breast cancer. Oncol. Lett..

[46] Lu, C., Wu, S., Liu, L. & Xing, D. Phototherapy-induced antitumor immunity: long-term tumor suppression effects via photoinactivation of respiratory chain oxidase-triggered superoxide anion burst. *Antioxidants Redox Signal.* **24**, (2016). 10.1089/ars.2015.6334 26413929

[47] Kobayashi, T. Antitumor effects of combined therapy of recombinant heat shock protein 70 and hyperthermia using magnetic nanoparticles in an experimental subcutaneous murine melanoma. *Cancer Immunol. Immunother.* 26–32 (2004) 10.1007/s00262-003-0416-5. 10.1007/s00262-003-0416-5 PMC11032910 14551746

[48] Zininga, T., Ramatsui, L. & Shonhai, A. Heat shock proteins as immunomodulants. *Molecules* **23**, (2018). 10.3390/molecules23112846 PMC6278532 30388847

[49] O’Shea J, Paul WE (2010). Mechanisms underlying lineage commitment and plasticity of helper CD4 + T cells. Science 80.

[50] Dobrzanski, M. J. Expanding roles for CD4 T cells and their subpopulations in tumor immunity and therapy. *Front. Oncol.* **3**, (2013). 10.3389/fonc.2013.00063 PMC3607796 23533029

[51] Ostroumov D, Fekete-Drimusz N, Saborowski M, Kühnel F, Woller N (2018). CD4 and CD8 T lymphocyte interplay in controlling tumor growth. Cell. Mol. Life Sci..

[52] Terunuma, H. Potentiating immune system by hyperthermia. in *Hyperthermic Oncology from Bench to Bedside* (eds. Kokura, S., Yoshikawa, T. & Ohnishi, T.) 127–135 (Springer, Singapore, 2016). 10.1007/978-981-10-0719-4_12.

[53] Zhou F (2018). Local phototherapy synergizes with immunoadjuvant for treatment of pancreatic cancer through induced immunogenic tumor vaccine. Clin. Cancer Res..

[54] Mojic M, Takeda K, Hayakawa Y (2018). The dark side of IFN-γ: Its role in promoting cancer immunoevasion. Int. J. Mol. Sci..

[55] Jarnicki, A. G., Lysaght, J., Todryk, S. & Mills, K. H. G. Suppression of Antitumor Immunity by IL-10 and TGF-␤-Producing T Cells Infiltrating the Growing Tumor: Influence of Tumor Environment on the Induction of CD4+ and CD8+ Regulatory T Cells. 2–10 (2006). 10.4049/jimmunol.177.2.896 16818744

[56] Plitas, G. & Rudensky, A. Y. Regulatory T Cells in Cancer. (2020).

[57] Romagnoli, G. *et al.* Morphological evaluation of tumor-infiltrating lymphocytes (TILs) to investigate invasive breast cancer immunogenicity, reveal lymphocytic networks and help relapse prediction: A retrospective study. *Int. J. Mol. Sci.* **18**, (2017). 10.3390/ijms18091936 PMC5618585 28885584

[58] Takada K (2018). Use of the tumor-infiltrating CD8 to FOXP3 lymphocyte ratio in predicting treatment responses to combination therapy with pertuzumab, trastuzumab, and docetaxel for advanced HER2-positive breast cancer. J. Transl. Med..

[59] Muthana M, Multhoff G, Pockley AG (2010). Tumour infiltrating host cells and their significance for hyperthermia. Int. J. Hyperth..

