# Supplementary material for: Hyperthermia by near infrared radiation induced immune cells activation and infiltration in breast tumor
Source: Sci Rep. 2021 May 13;11:10278. doi: 10.1038/s41598-021-89740-0 (PMC8119485; doi:10.1038/s41598-021-89740-0)
Supplement: Supplementary file 1 — Supplementary Information. [file 41598_2021_89740_MOESM1_ESM.docx]

**Supplementary Information**

Hyperthermia by Near Infrared Radiation Induced Immune Cells Activation and Infiltration in Breast Tumor

W. F. A. Wan Mohd Zawawi, M. H. Hibma, M. I. Salim and K. Jemon


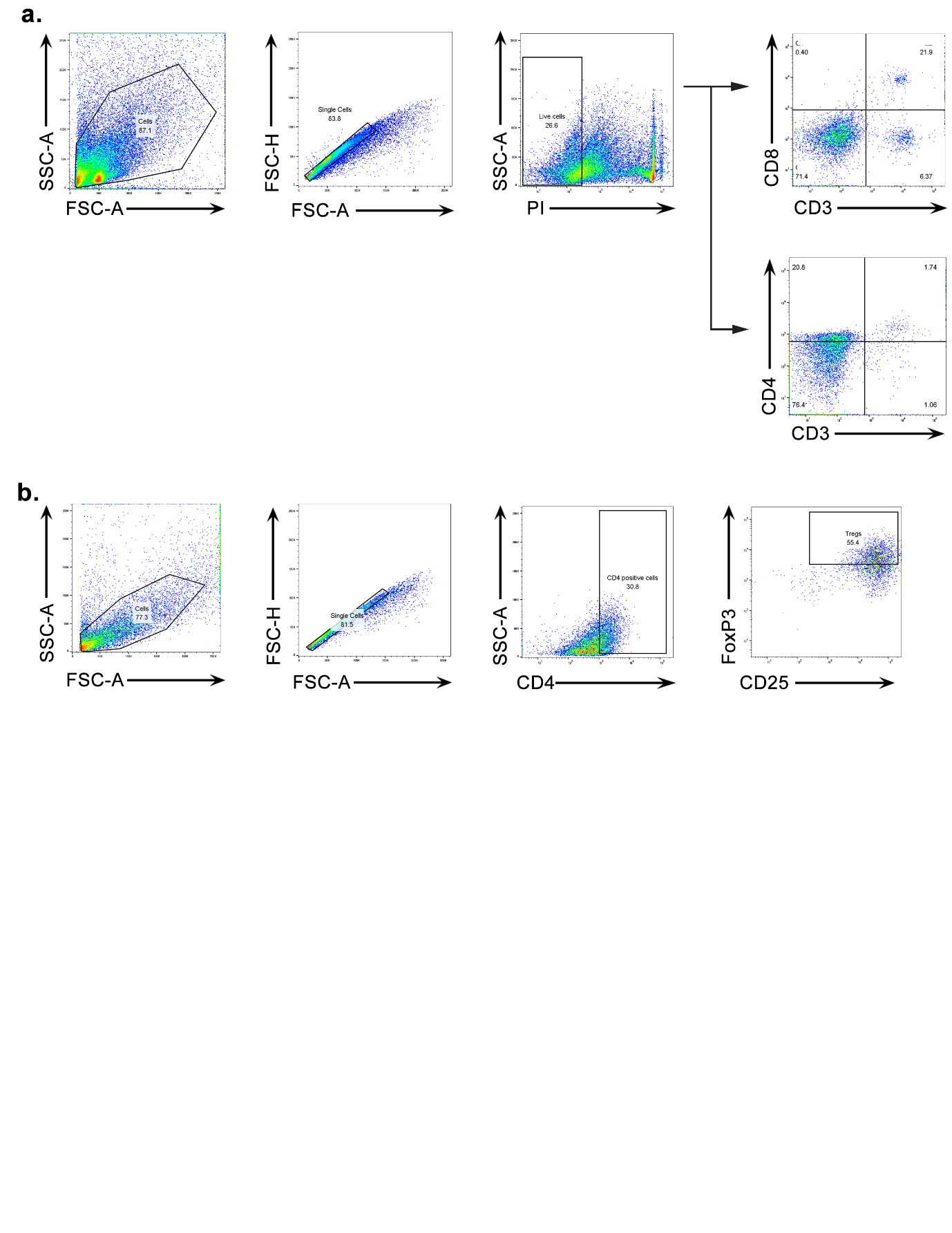


**Supplementary Figure 1.** Dot plot showing the gating strategy of **(a)** CD4^+^ and CD8^+^ T cells and **(b)** Tregs. Cells were gated based on forward and side scatter profile for doublet exclusion. PI live/dead staining was performed to remove dead cells.
